# Drug resistance profile and biofilm forming potential of *Pseudomonas aeruginosa* isolated from contact lenses in Karachi-Pakistan

**DOI:** 10.1186/1471-2415-13-57

**Published:** 2013-10-17

**Authors:** Syed H Abidi, Sikandar K Sherwani, Tarrunum R Siddiqui, Asma Bashir, Shahana U Kazmi

**Affiliations:** 1 Immunology and Infectious Diseases Research Lab, Department of Microbiology, University of Karachi, Karachi, Pakistan; 2 Department of Biological and Biomedical Sciences, Aga Khan University, Stadium Road, Karachi 74800, Pakistan; 3 Department of Microbiology, Federal Urdu University of Arts, Science and Technology, Karachi, Pakistan; 4 Akhtar Eye Hospital, and Pakistan Medical Research Council, Karachi, Pakistan; 5 Department of Biosciences, Shaheed Zulfikar Ali Bhutto Institute of Science and Technology (SZABIST), Karachi, Pakistan

**Keywords:** Antibiogram, Biofilm, Pseudomonas aeruginosa, Contact lens

## Abstract

**Background:**

The contaminated contact lens provides *Pseudomonas aeruginosa* an ideal site for attachment and biofilm production. Continuous contact of the eye to the biofilm-infested lens can lead to serious ocular diseases, such as keratitis (corneal ulcers). The biofilms also prevent effective penetration of the antibiotics, which increase the chances of antibiotic resistance.

**Methods:**

For this study, 22 *Pseudomonas aeruginosa* isolates were obtained from 36 contact lenses and 14 contact lens protective fluid samples. These isolates were tested against eight commonly used antibiotics using Kirby-Bauer disk diffusion method. The biofilm forming potential of these isolates was also evaluated using various qualitative and quantitative techniques. Finally, a relationship between biofilm formation and antibiotic resistance was also examined.

**Results:**

The isolates of *Pseudomonas aeruginosa* tested were found resistant to most of the antibiotics tested. Qualitative and quantitative biofilm analysis revealed that most of the isolates exhibited strong biofilm production. The biofilm production was significantly higher in isolates that were multi-drug resistant (p < 0.0001).

**Conclusion:**

Our study indicates that multi-drug resistant, biofilm forming *Pseudomonas aeruginosa* isolates are mainly involved in contact lens associated infections. This appears to be the first report from Pakistan, which analyzes both antibiotic resistance profile and biofilm forming potential of *Pseudomonas aeruginosa* isolates from contact lens of the patients with contact lens associated infections.

## Background

*Pseudomonas aeruginosa* is a clinically significant pathogen involved in several important infections, such as nosocomial, respiratory tract, urinary tract, burns, wound, and eye, etc. [1-4]. One of the hallmarks of Pseudomonal infection is its capability to adhere to and propagate on medical devices like *catheters*, contact lenses etc. The adherence is aided by several microbial factors, in which biofilm formation holds a key position [4,5]. Contact lens is one such devise that is frequently used for medical or cosmetic purpose. Improper handling and unhygienic use of the contact lens allows pathogens, such as *Pseudomonas aeruginosa,* to adhere to and produce biofilm on the surface of the lens [6]. Continuous contact of the eye to the biofilm-infested lens can result in serious eye infections such as keratitis (corneal ulcers) [7-10], which, if left untreated, can ultimately lead to vision loss. The improper management of these infections not only cause serious eye problems, but also increase the chances for antibiotic resistance [11].

In countries like Pakistan, contact lenses, of varying price and quality, can be purchased easily without any authentic prescription. Most of the users are unaware of proper hygienic practices, and as a result, they often face contact lens associated ophthalmic complications. In Pakistan, there are no significant details available about the burden of contact lens associated infection by *Pseudomonas aeruginosa* and antimicrobial resistance profile in such cases. In this study, we analyzed the antibiotic susceptibility/resistance profile and biofilm forming potential of *Pseudomonas aeruginosa* – isolated from patients with contact lens associated infections. Furthermore, we also examined the relationship between *Pseudomonas aeruginosa* biofilm forming potential and antibiotic resistance profile.

## Methods

### a) Sample collection, isolation and identification of cultures

For this study, a total of 36 contact lenses, as well as 14 samples of contact lens protective fluid, were collected from Akhter Eye Hospital, Karachi-Pakistan. The contact lenses were aseptically immersed into Brain Heart Infusion (BHI) broth (Sigma-Aldrich) tubes and vortexed for 1 minute. Subsequently, the lenses were removed and the BHI broth tubes were incubated at 37°C for 24 hours. After incubation, a loop full of broth was inoculated on the Nutrient Agar (Sigma-Aldrich) plates and the plates were incubated at 37°C for 24 hours. For contact lens fluid samples, a loop full of liquid was directly inoculated and smeared on the Nutrient Agar (Sigma-Aldrich) plates and the plates were incubated at 37°C for 24 hours. The cultures were identified by using conventional and rapid biochemical tests (Quick test strip 24, DESTO, Pakistan).

### b) Antibiogram development

Antibiotic susceptibility/resistance patterns of *Pseudomonas aeruginosa* to Vancomycin (VA), Erythromycin (E), Tetracycline (TE), Chloramphenicol (C), Ampicillin (AMP), Ofloxacin (OFX), Cephalexin (CL) and Gentamicin (CN) was examined by Kirby-Bauer method [12]. Briefly, 0.5 McFarland (10^8^ cfu/ml) *Pseudomonas aeruginosa* inoculum was smeared on the Nutrient Agar plates, and above-mentioned antibiotics discs were placed onto the agar. The plates were incubated at 37°C for 24–48 hours, after which, the zone of inhibition was measured.

### c) Evaluation of biofilm forming potential

The biofilm forming potential of *Pseudomonas aeruginosa* was evaluated by following methods: 1) Tube method, 2) Air Liquid Interface Assay, and 3) Microtitre-plate method.

#### Tube method

The tube method was performed in two different ways [13]. In the first experiment the cultures of *Pseudomonas aeruginosa* were inoculated in 3–5 ml Tryptone Soy Broth (TSB; Sigma-Aldrich) tubes and inoculated at 37°C for 48 hours. After incubation, the biofilm formation (dense matt formation) was observed at the air-liquid interface of the tubes. In the second experiment, cultures of *Pseudomonas aeruginosa* were inoculated in 3–5 ml Tryptone Soy Broth (TSB; Sigma-Aldrich) tubes and incubated at 37°C for 48 hours. After incubation, the content of the tubes were decanted and the tubes were washed with Phosphate Buffer Saline (PBS; pH 7) and left to air-dry. Subsequently, the tubes were stained with Crystal Violet (0.1% w/v), and the tubes were gently rotated to ensure uniform staining. Afterwards, the stain was removed and tubes were washed with sterile distilled water, and then dried in inverted position. Biofilm formation was considered positive when a visible stained film was observed adhered to the wall and bottom of the tube. Tubes were scored as: 0-absent, 1-weak, 2-moderate or 3-strong. Experiments were performed in triplicate and repeated three times.

#### Air liquid interface assay

The *Pseudomonas aeruginosa* biofilms were microscopically visualized using Air liquid interface assay [14]. The assay was performed in two different ways. In first experiment, cultures of *Pseudomonas aeruginosa* were 1:100 diluted in 3 ml Tryptone Soy Broth (TSB; Sigma-Aldrich). Subsequently, 300 μl of each diluted culture was pipetted into each well of a 12-well, flat-bottom plates. The plates were covered with lid and incubate at 37°C for 48 hour, in a position that they were making a 45° angle to the surface of incubator. After incubation, the cultures were aspirated and the wells were gently washed twice by adding 400 μl sterile TSB medium. After two washes, 200 μl of TSB medium was added to each well. The plate was laid flat on the stage of an inverted microscope (Olympus, Japan) and biofilm formation was visualized. The pictures of the biofilm were taken using a digital camera (Canon-A450, Malaysia).

In the second experiment, cultures of *Pseudomonas aeruginosa* were 1:100 diluted in 3 ml Tryptone Soy Broth (TSB; Sigma-Aldrich). Subsequently, 300 μl of each diluted culture was inoculated in each well of a 12-well plate with cover slips placed at 90^o^ angle to the well’s surface. The 12-well plate was incubated for 48 hours at 37°C. After incubation, cover slips were taken out, washed with sterile distill water and stained with 0.1% CV for 10 minutes. Afterwards, the cover slips were washed and dried. Dry cover slips were visualized under high power microscope (Olympus, Japan) and pictures were taken using a digital camera (Canon-A450, Malaysia).

#### Microtitre-plate method

Finally, the *Pseudomonas aeruginosa* cultures were quantitatively analyzed for their biofilm forming potential using microtitre-plate method [14-16]. Briefly, *Pseudomonas aeruginosa* cultures were inoculated in 3–5 ml TSB and incubated for 24 hours at 37°C. After incubation, cultures were 1:100 diluted in the TSB, and 100 μl of each diluted culture was pipetted in each well of 96-well flat-bottom microtiter plate (non-tissue culture treated, Sigma-Aldrich). Plates was covered and incubated at 37°C for 48 hours. After incubation, contents of the well were aspirated out and the wells were washed thoroughly with PBS. Subsequently, the wells were stained for 10 min by adding 125 ul of 0.1% Crystal Violet (w/v) solution to each well. Afterwards, the stain was removed and the plate was washed with clean tap water and left to air dry. Subsequently, 200 μl of 95% Ethanol was added to each stained well and plates were incubated for 10 to 15 min at room temperature. Contents of each well were mixed by pipetting, and then 125 μl of the Crystal Violet/Ethanol solution was transferred from each well to a separate well of an optically clear flat-bottom 96-well plate. Optical densities (OD) of each of these 125-μl samples were measured at 630 nm using spectrophotometer (Starfax 2100, Awareness Technology Inc). Experiments were performed in duplicate.

### d) Analysis of relationship between biofilm formation and antibiotic resistance

In order to examine the relationship between forming potential and antibiotic resistance, the antibiotic susceptible and antibiotic resistant isolates were separately grouped and biofilm forming potential of each group was examined. The difference between the groups was analyzed using unpaired two-tailed student’s T test using GraphPad software with significance level of p < 0.05.

## Results

### Isolation of *Pseudomonas aeruginosa* from contact lenses and contact lens solutions

A total of 22 *Pseudomonas aeruginosa* isolates were obtained from 36 contact lenses and 14 contact lens solution samples. The cultures were identified on the basis of colony morphology, pigment production and characteristic biochemical reactions using both conventional and rapid identification tests (Quick strip).

### Antibiotic resistance/susceptibility profile of *Pseudomonas aeruginosa* isolates

**Figure 1:**
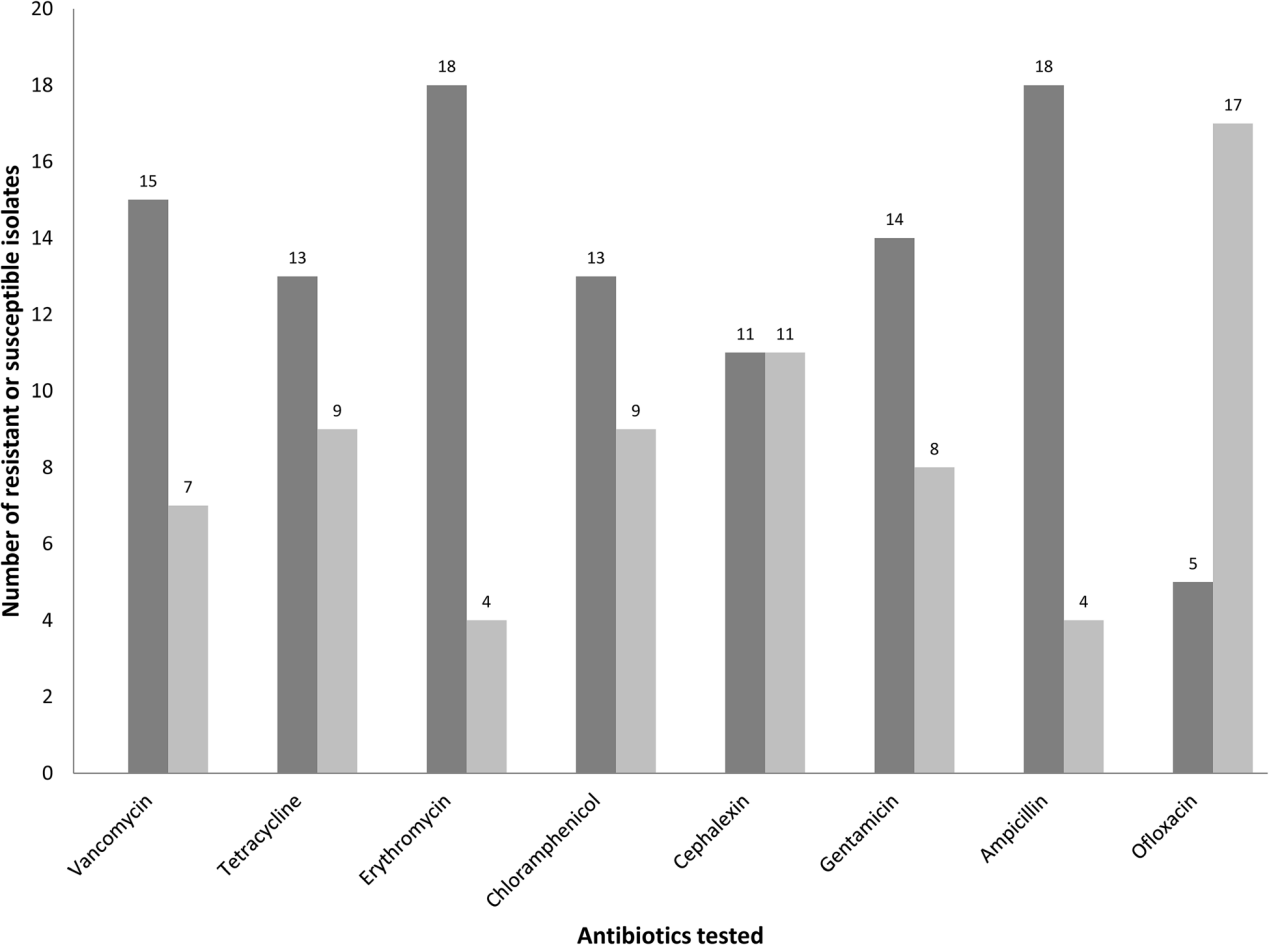
**Antibiogram for *Pseudomonas aeruginosa *isolates*.*** Antibiotic resistance or susceptibility profile was developed by testing *Pseudomonas aeruginosa* isolates against eight commonly prescribed antibiotics. Light grey bar represents number of susceptible pathogens while dark grey bar shows number of resistant pathogens.

The antibiotic resistance/susceptibility profile of Pseudomonas isolates revealed that most of the isolates were resistant to one or more tested antibiotics (Figure 1). Most of the isolates (18/22) were resistant to Erythromycin and Ampicillin (Figure 1). Cephalexin and Ofloxacin were found to be the most effective antibiotics as respectively, 11/22 and 17/22 isolates were susceptible to them (Figure 1).

### Biofilm forming potential of *Pseudomonas aeruginosa* isolates

**Figure 2:**
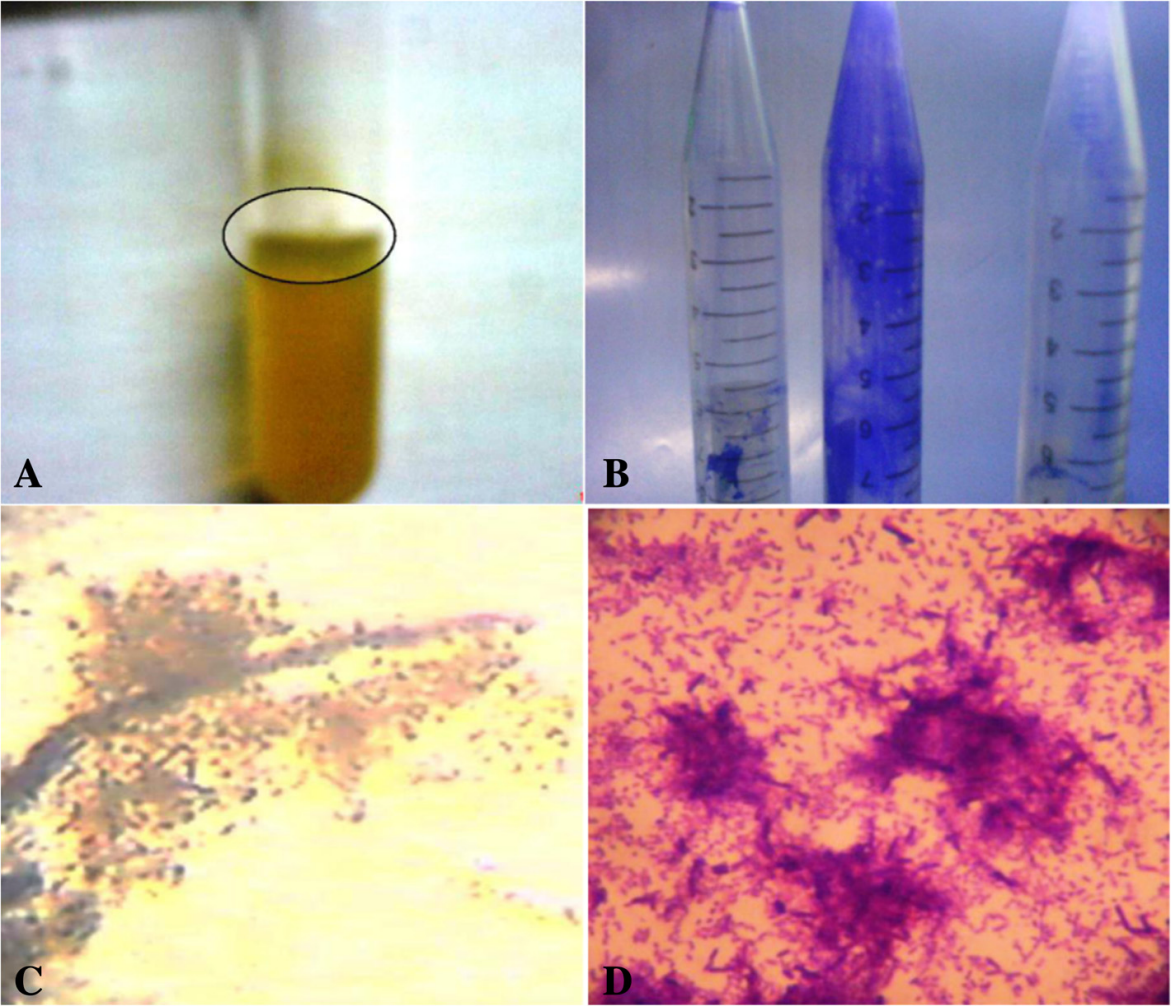
**Qualitative analysis of biofilm formation by *Pseudomonas aeruginosa*.** Pictures showing **A)** dense matt formed at the Air-liquid interface in glass tube (pointed by arrow), **B)** microorganisms adhered to the surface of polystyrene surface stained with Crystal Violet. **A and B)** The experiment was performed using tube method. Biofilm formation by *Pseudomonas aeruginosa* as observed **C)** under inverted microscope using Air-liquid interface assay, **D)** under compound microscope using Air-Liquid interface cover slip assay. Dense matt formation and microbial aggregation is clearly evident in the **C)** wells and **D)** on the slides.

**Figure 3:**
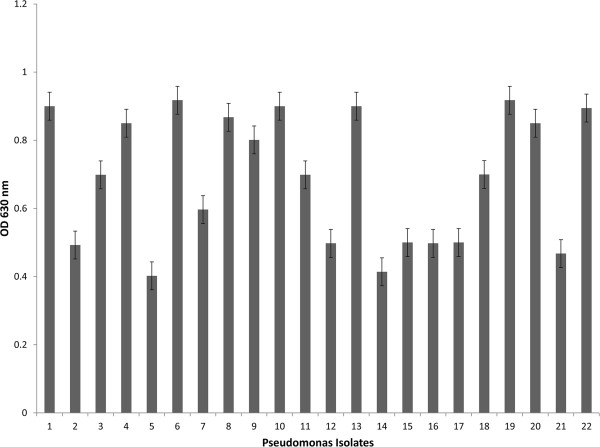
**Quantitative analysis of biofilm formation by *Pseudomonas aeruginosa*.** Graph showing different OD obtained for each isolate as calculated by 96- well microtitre plate assay. The experiment was performed in duplicate and the error bars represents standard error of their mean.

In this study, the biofilm forming potential of *Pseudomonas aeruginosa* isolates was evaluated using both qualitative (tube method, air liquid interface assay) and quantitative (micro-titre plate assay) methods. In the tube method, *Pseudomonas aeruginosa* isolates, inoculated in the tubes, formed a dense whitish matt at air-liquid Interface (Figure 2A) and also strongly adhered to the walls of the tubes (Figure 2B). Similarly, the results for the Air liquid assay revealed that the culture of *Pseudomonas aeruginosa* exhibited dense matt formation (Figure 2C) and strong aggregation (Figure 2D). The quantitative biofilm analysis corroborated the qualitative results, where almost all *Pseudomonas aeruginosa* isolates were found to be dominant biofilm former with OD_630_ >0.5 (Figure 3).

### Relationship between biofilm formation and antibiotic resistance profile

The statistical analysis to examine the link between antibiotic resistance and biofilm formation showed that the biofilm production in multi-drug resistant isolates was significantly higher that drug susceptible isolates (P < 0.0001).

## Discussion

*Pseudomonas aeruginosa* has emerged as an important eye pathogen, responsible for serious ophthalmic infections such as keratitis (corneal ulcers) [17,18]. Increasing use of contact lens – both for medical or cosmetic use – has greatly increased the risk for acquiring Pseudomonal infections [19]. Improper handling and use of contaminated storage solution can pollute the contact lens, which in turn serve as an ideal platform for the bacterial adherence and biofilm production [20,21]. The biofilm forming potential has been associated with increased antibiotic resistance, which ultimately leads to therapeutic failure [22,23]. Analysis of the susceptibility/resistance profile our *Pseudomonas aeruginosa* isolates reveled that majority of them were resistant to one or more tested antibiotics. Pinna *et al.*, and Ly *et al.*, found Aminoglycosides and Fluoroquinolones to be effective in contact lens associated Pseudomonal infections [19,21,24], while Wilcox reported first generation of Cephalosporins with Aminoglycosides as an effective initial treatment in contact lens associated Pseudomonal infections [25]. Our findings were in agreement with these reports, where Pseudomonas isolates exhibited lowest resistance against Ofloxacin (Fluoroquinolone) and Cephalexin (first generation Cephalosporins). However, in contrast to above-mentioned studies, high resistance was observed against Gentamycin (aminoglycoside). In the next step, we analyzed the biofilm forming potential of these isolates, as biofilm plays important role in antimicrobial resistance [26]. Analysis of biofilm formation potential, using both qualitative and quantitative methods, identified almost all *Pseudomonas aeruginosa* isolates as dominant biofilm formers. Finally, the analysis for relationship between biofilm formation and antibiotic resistance/susceptibility revealed that the multi-drug resistant isolates displayed significant biofilm production as compared to susceptible isolates (p < 0.0001). Our results are consistent with previous reports, where the minimum inhibitory concentration of different antibiotics in different microbial biofilms were found to increase from 10–1000 fold, when compared to non-biofilm forming colonies [27]. This resistance can be explained by the three possible mechanisms: 1) Failure of the antibiotics to penetrate the dense matrix, 2) Sub-optimal concentration of antibiotic, in case the antibiotic penetrates the biofilm, which is below the minimum inhibitory concentration for the microbes inside the biofilm, 3) Inability of the antibiotic to inhibit pathogens, because most the pathogens in deeper layers of biofilm are metabolically inactive, and 4) Expulsion of antibiotics from the biofilm, as a result of cumulative ‘efflux action’ by the microbial communities [22].

We believe our study would serve as a first significant report on Pakistani patients, analyzing the status of antibiotic resistance and biofilm formation in *Pseudomonas aeruginosa,* isolated from contact lens of patients with different ocular infections. Our results strongly emphasize on the importance of: 1) Proper handling and hygienic storage of contact lens in order to prevent Pseudomonal infections, 2) Selecting correct and effective antimicrobial agents in ocular infections to prevent and control antimicrobial resistance, and 3) Exploring new antimicrobial agents that can target biofilms producing pathogens, especially *Pseudomonas aeruginosa*, that are involved in contact lens associated infections.

## Conclusion

In conclusion, observing hygienic practices while dealing with contact lenses can save the user from Pseudomonal eye infections. Additionally, careful selection of appropriate antibiotics in contact lens associated infections can not only prevent serious ocular complications, but can also reduce antimicrobial resistance and dissemination of biofilm forming Pseudomonal isolates.

## Competing interests

The authors declared that they have no competing interests.

## Authors’ contributions

Study design and analysis: SHA, SKS, SUK. Experiments: SHA, SKS, TRS, AB. Manuscript writing: SHA, SUK. Supervision: SUK. Syed H. Abidi (SHA), Sikandar K. Sherwani (SKS), Tarrunum R. Siddiqui (TRS), Asma Bashir (AB) and Shahana U. Kazmi (SUK). All authors read and approved the final manuscript.

## Pre-publication history

The pre-publication history for this paper can be accessed here:

http://www.biomedcentral.com/1471-2415/13/57/prepub
